# Supplementary material for: Structure of the plastic-degrading Ideonella sakaiensis MHETase bound to a substrate
Source: Nat Commun. 2019 Apr 12;10:1717. doi: 10.1038/s41467-019-09326-3 (PMC6461665; doi:10.1038/s41467-019-09326-3)
Supplement: Supplementary file 1 — Supplementary Information [file 41467_2019_9326_MOESM1_ESM.pdf]

Structure of the plastic-degrading *Ideonella sakaiensis* MHETase bound to a substrate

G.J. Palm, L. Reisky *et al.*

Supplementary Figure 1

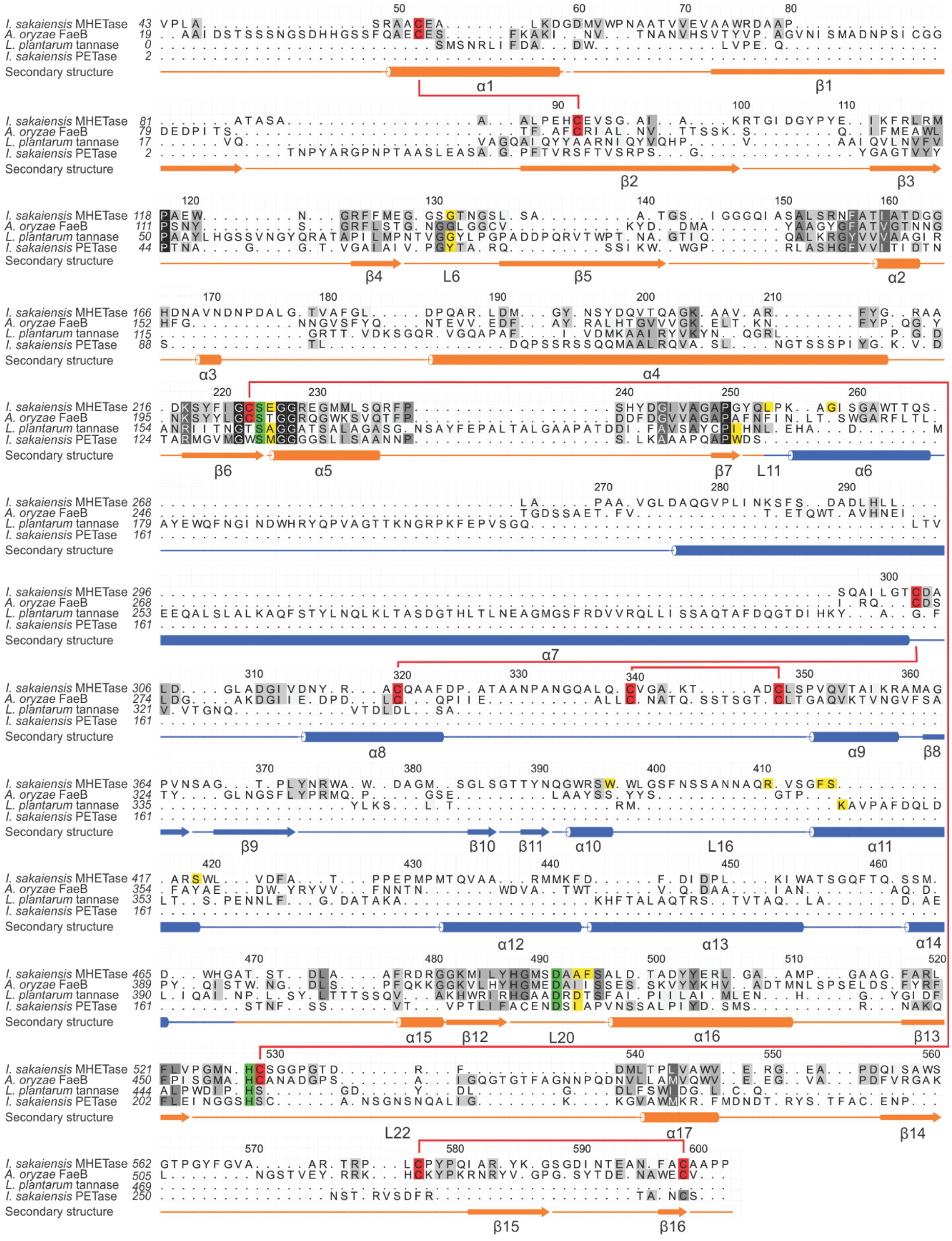

Supplementary Figure 1 Structure-based alignment of *I. sakaiensis* MHETase, PETase, *A. oryzae* feruloyl esterase (FaeB, PDB-ID: 3WMT[1]) and *L. plantarum* acyl tannase (PDB-ID: 4J0K [2]). The alignment was prepared by Chimera employing Clustal Omega[3] and shaded with ALSCRIPT[4]. Proteins are identified on the left of the aligned sequences. Higher conservation is indicated by a darker background. Numbering refers to the respective proteins. Below the alignment, secondary structure elements (α – α-helix, β – β-sheet, L – loop) of MHETase are shown in orange (α/β-hydrolase domain) and marine blue (lid domain) and numbered, a dashed line indicates residues absent from the MHETase structure. Yellow shading indicates residues which contribute to substrate binding, green shading residues of the catalytic triad, red shading and connecting red lines cysteines involved in disulfide bonds.

Supplementary Figure 2

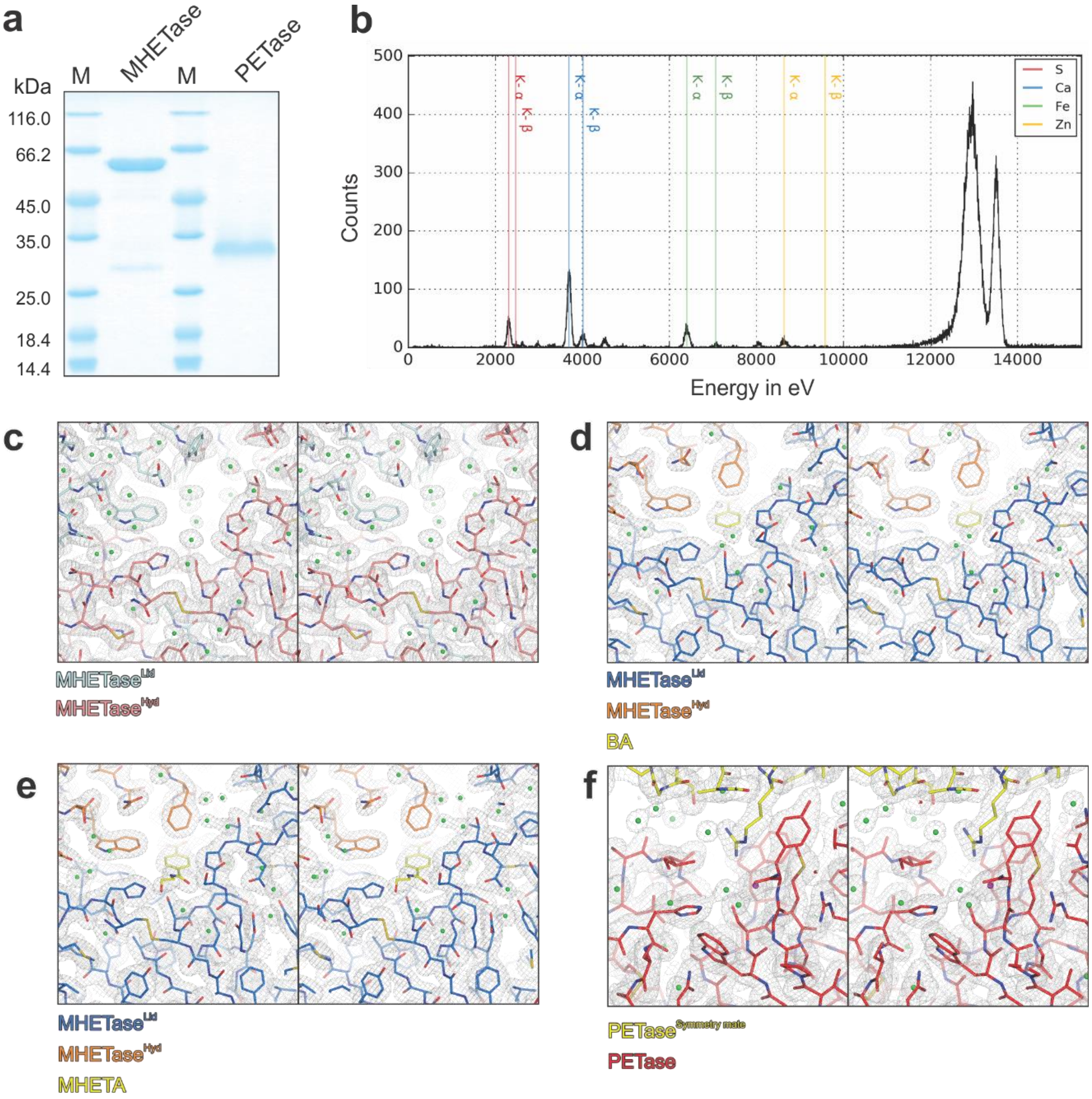

Supplementary Figure 2 PETase and MHETase sample quality, X-ray fluorescence spectrogram of MHETase crystals and refined electron densities. **(a)** SDS-PAGE analysis of purified PETase and MHETase. M – Marker **(b)** X-ray fluorescence spectrogram of MHETase crystals displayed and analyzed using the program XFEPLOT (https://www.helmholtz-berlin.de/forschung/oe/np/gmx/xfeplot/index_en.html). A crystal of MHETase was exposed to an X-ray beam of 13.5 keV energy on the HZB-MX beamline BL14.1 and the resulting fluorescence recorded using a fluorescence detector[5]. The Fe-signal is likely derived from the sample holder, the sample may also contain traces of Zn. **(c-f)** Refined $2F_O$-F_C electron-density maps (grey mesh) of **(c)** MHETase, **(d)** MHETase in complex with benzoic acid, **(e)** MHETase in complex with MHETA and **(f)** PETase. As observed in a prior study, in the crystal lattice of PETase the R105 side chain of a symmetry-related molecule inserts into the PETase active site[6].

Supplementary Figure 3

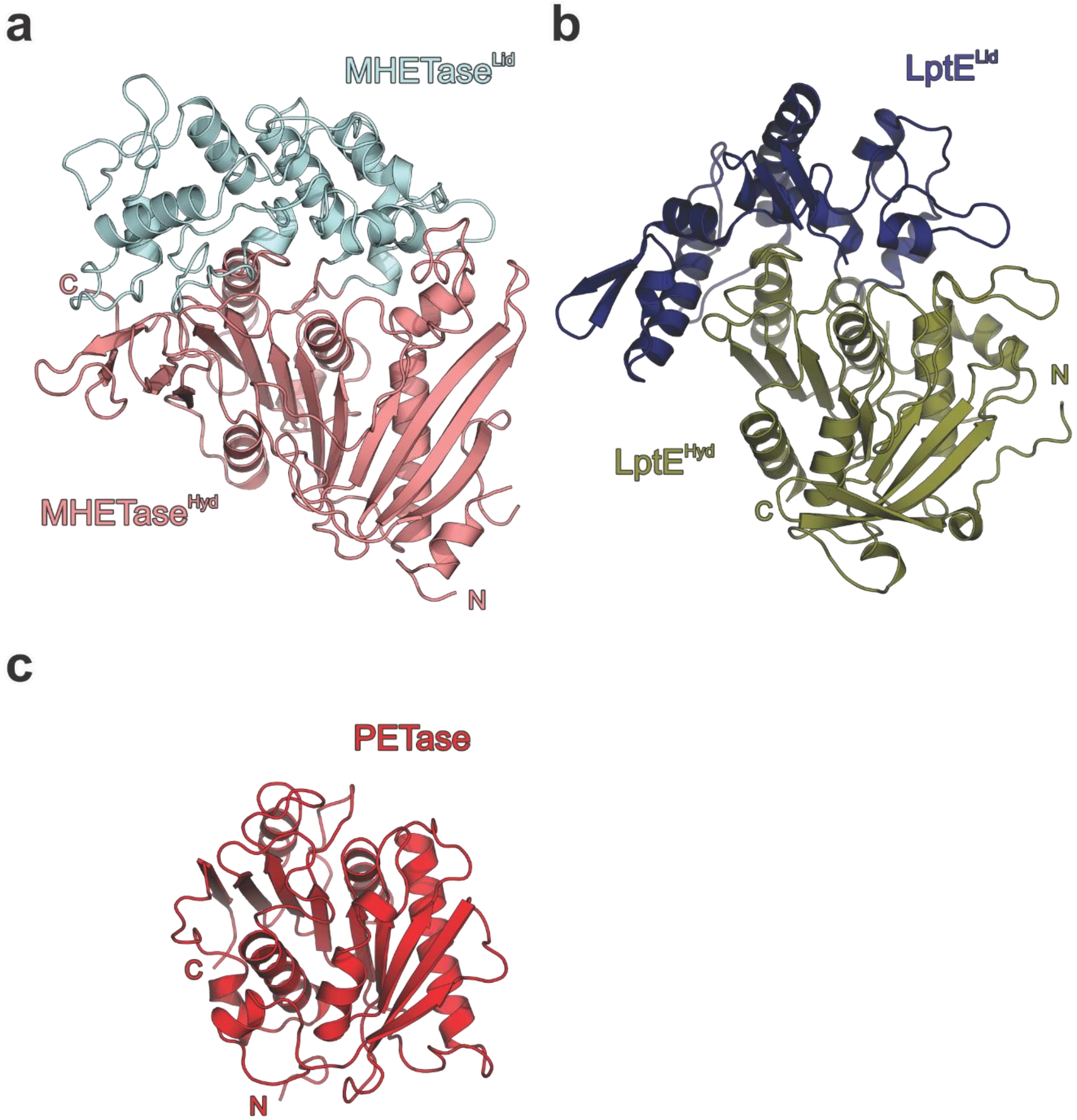

Supplementary Figure 3 Comparison of *I. sakaiensis* MHETase, PETase and tannin acyl α/β-hydrolase from *L. plantarum*. **(a-c)** Comparison of **(a)** MHETase with **(b)** tannin acyl α/β-hydrolase from *L. plantarum* (PDB-ID:4J0K[2]) and **(c)** PETase (red). α/β-Hydrolase and lid domains are colored as in Figures 1 and 2.

Supplementary Figure 4

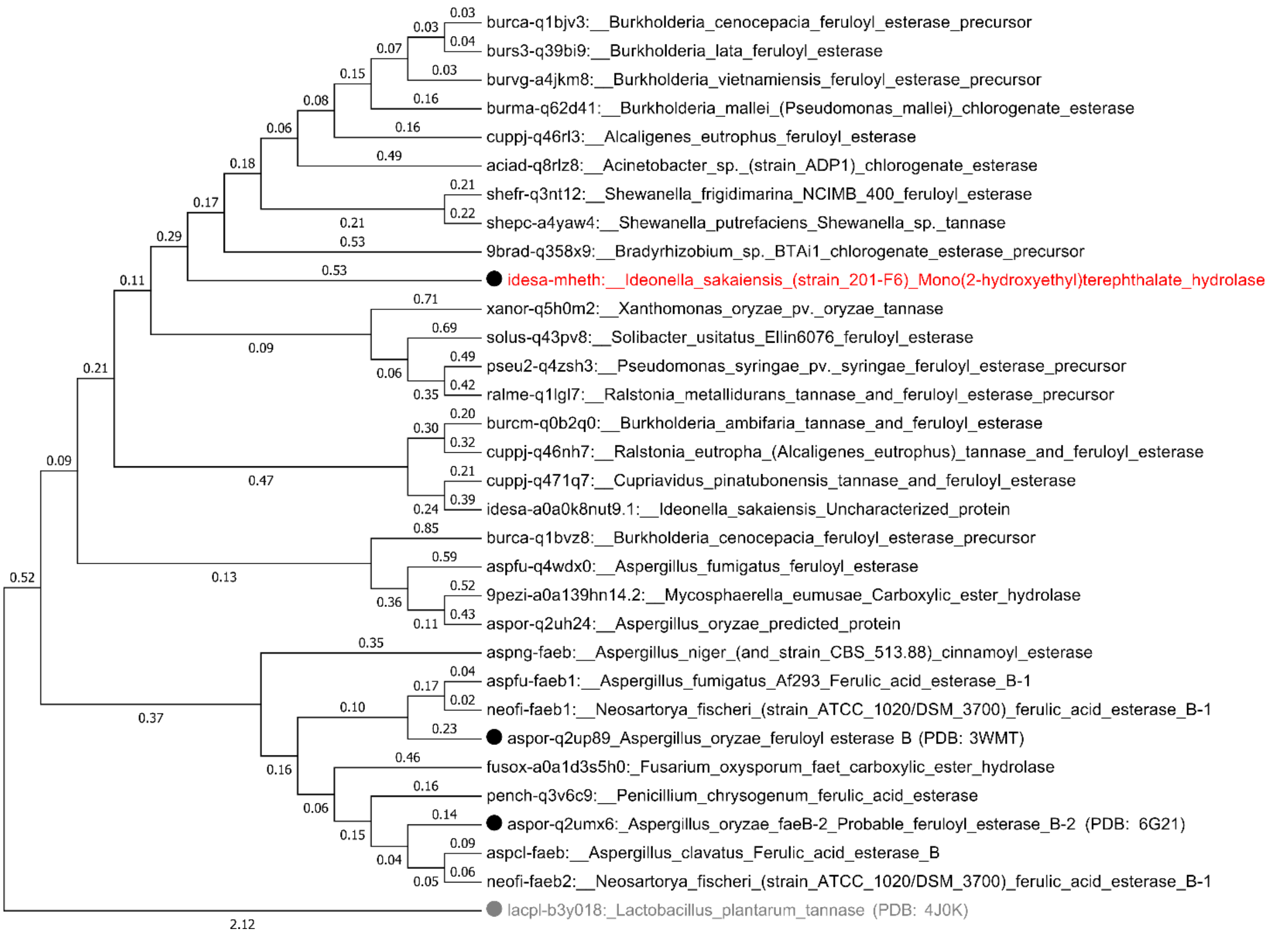

Supplementary Figure 4 Molecular phylogenetic analysis of feruloyl esterases/tannases by Maximum Likelihood method. Sequences were obtained from a BLAST search in the ESTHER Block_X.pep database. From this dataset 32 sequences with the highest similarity and with annotations were chosen. Multiple sequence alignment was performed by Muscle alignment using MEGA7. The tree was also calculated using MEGA7 with displayed branch lengths showing the evolutionary distances. Sequences with structural data are marked with a black circle. The sequence of the structurally related *L. plantarum* tannase from Block H of the bacterial tannases (grey) from the ESTHER database was added manually to the alignment and the tree in order to visualize the phylogenetic relationship.

Supplementary Figure 5

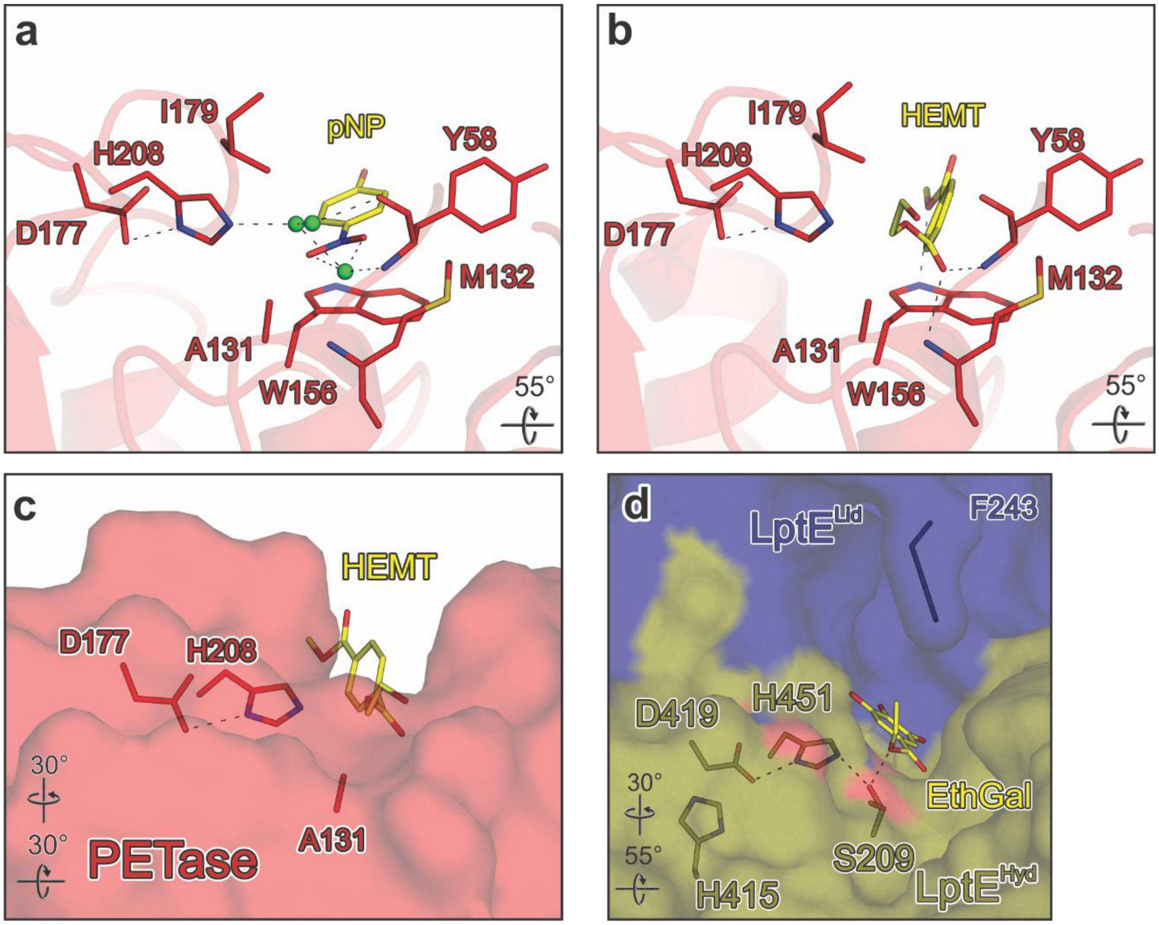

Supplementary Figure 5 Comparison of *I. sakaiensis* PETase and tannin acyl α/β-hydrolase from *L. plantarum*. α/β-Hydrolase and lid domains are colored as in Figures 1 and 2 **(a)** Close-up view of *I. sakaiensis* PETase (red; PDB-ID:5XH2[6]) bound to pNP (yellow), superimposed on helix α5 of MHETase. (**b)** Close-up view of *I. sakaiensis* PETase (red; PDB-ID:5XH3[6] bound to HEMT (yellow), superimposed on helix α5 of MHETase. **(c)** Close-up view of the PETase substrate binding site shown as molecular surface (red; PDB-ID:5XH3[6]) bound to HEMT (yellow). The catalytic triad residues are shown as sticks. **(c)** Molecular surface of the LptE (PDB-ID:4J0K[2]) active site bound to ethyl gallate (EthGal, yellow). The catalytic triad residues, H415 and F243 are shown as sticks. Rotation symbols indicate views relative to Fig. 1b.

Supplementary Figure 6

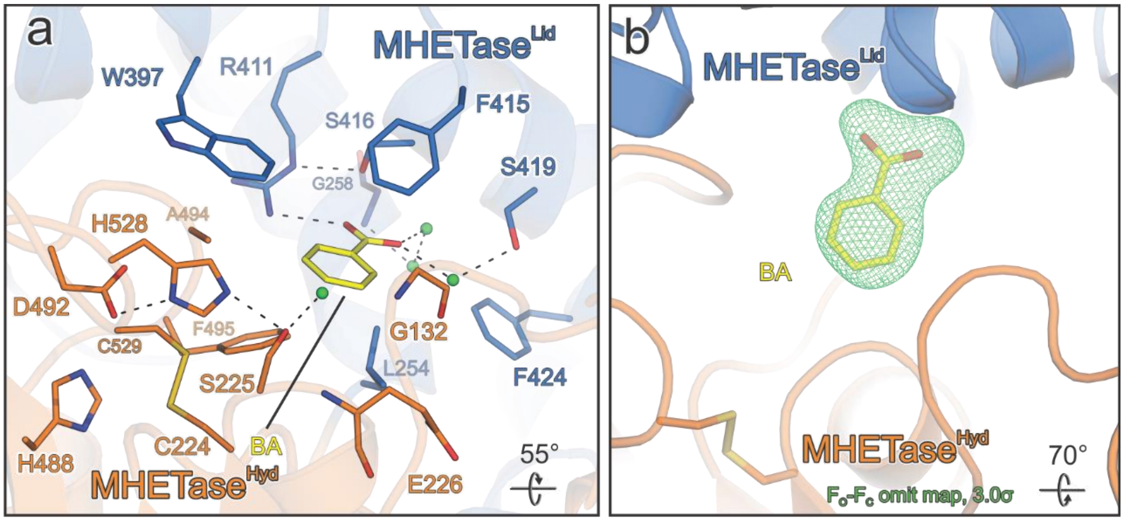

Supplementary Figure 6 Structure of MHETase in complex with benzoic acid rationalizes substrate requirements **(a)** Close-up view on the active site of MHETase (MHB) bound to benzoic acid (BA, yellow). **(b)** Refined F_OF_C-omit density (green) contoured at 3σ for benzoic acid. Benzoic acid of the refined final structure is shown as sticks. Colors and labels as in Fig. 2b. Rotation symbols indicate views relative to (Fig. 1b)

Supplementary Figure 7

a

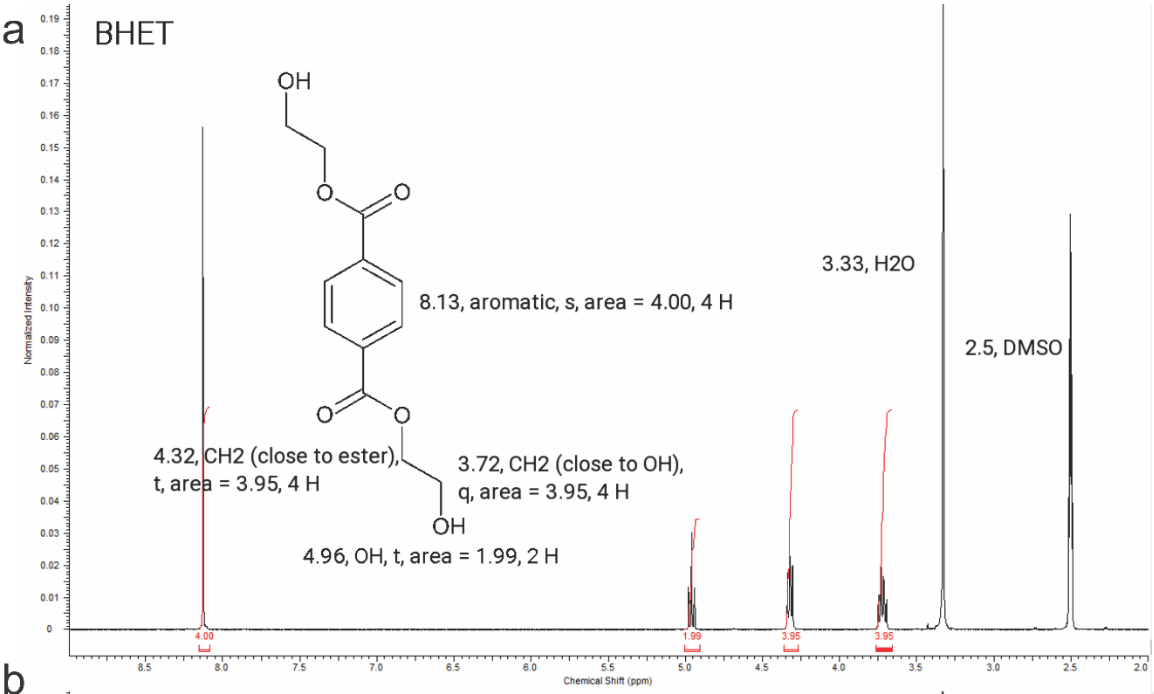

b

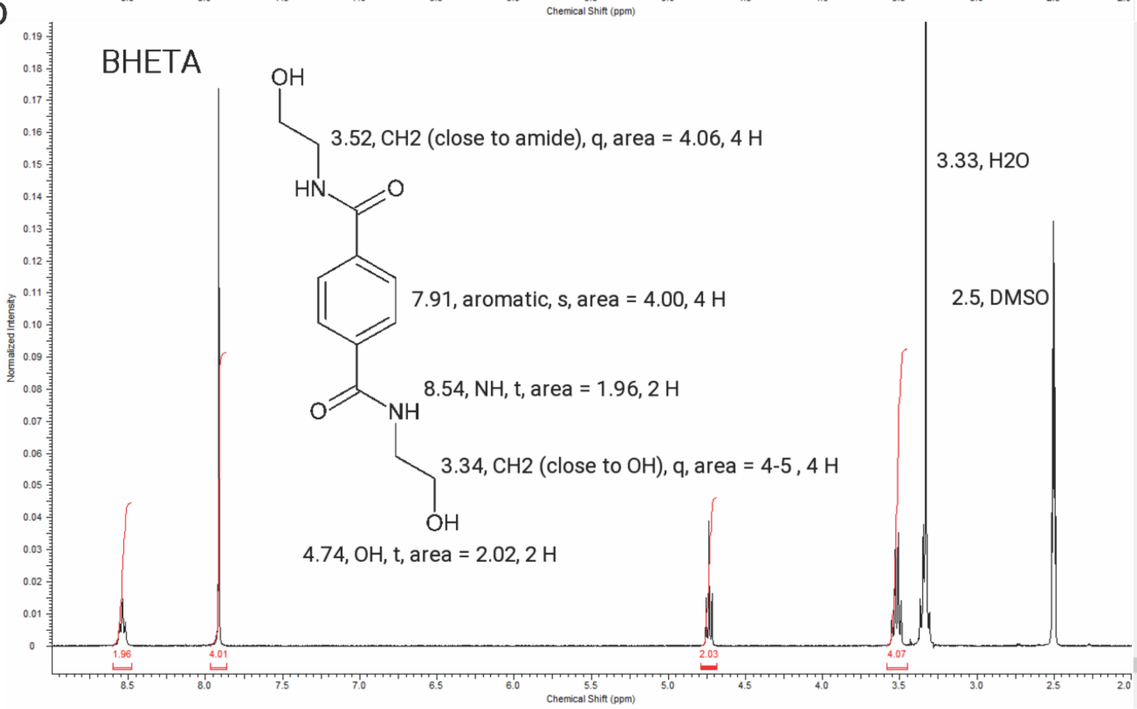

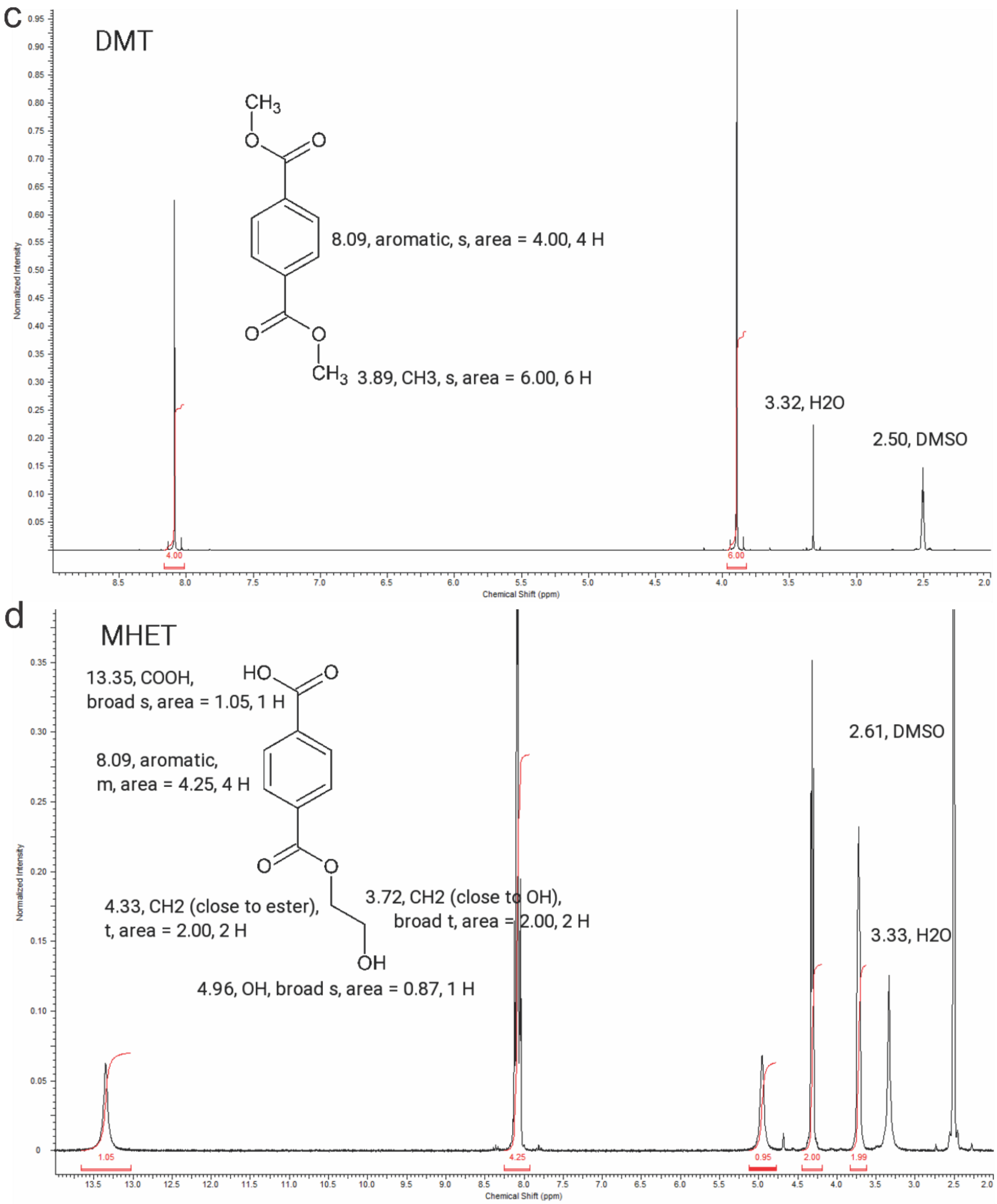
c
DMT
8.09, aromatic, s, area = 4.00, 4 H
3.89, CH3, s, area = 6.00, 6 H
3.32, H2O
2.50, DMSO
Normalized Intensity
Chemical Shift (ppm)
d
MHET
13.35, COOH,
broad s, area = 1.05, 1 H
8.09, aromatic,
m, area = 4.25, 4 H
4.33, CH2 (close to ester),
t, area = 2.00, 2 H
3.72, CH2 (close to OH),
broad t, area = 2.00, 2 H
4.96, OH, broad s, area = 0.87, 1 H
2.61, DMSO
3.33, H2O
Normalized Intensity
Chemical Shift (ppm)

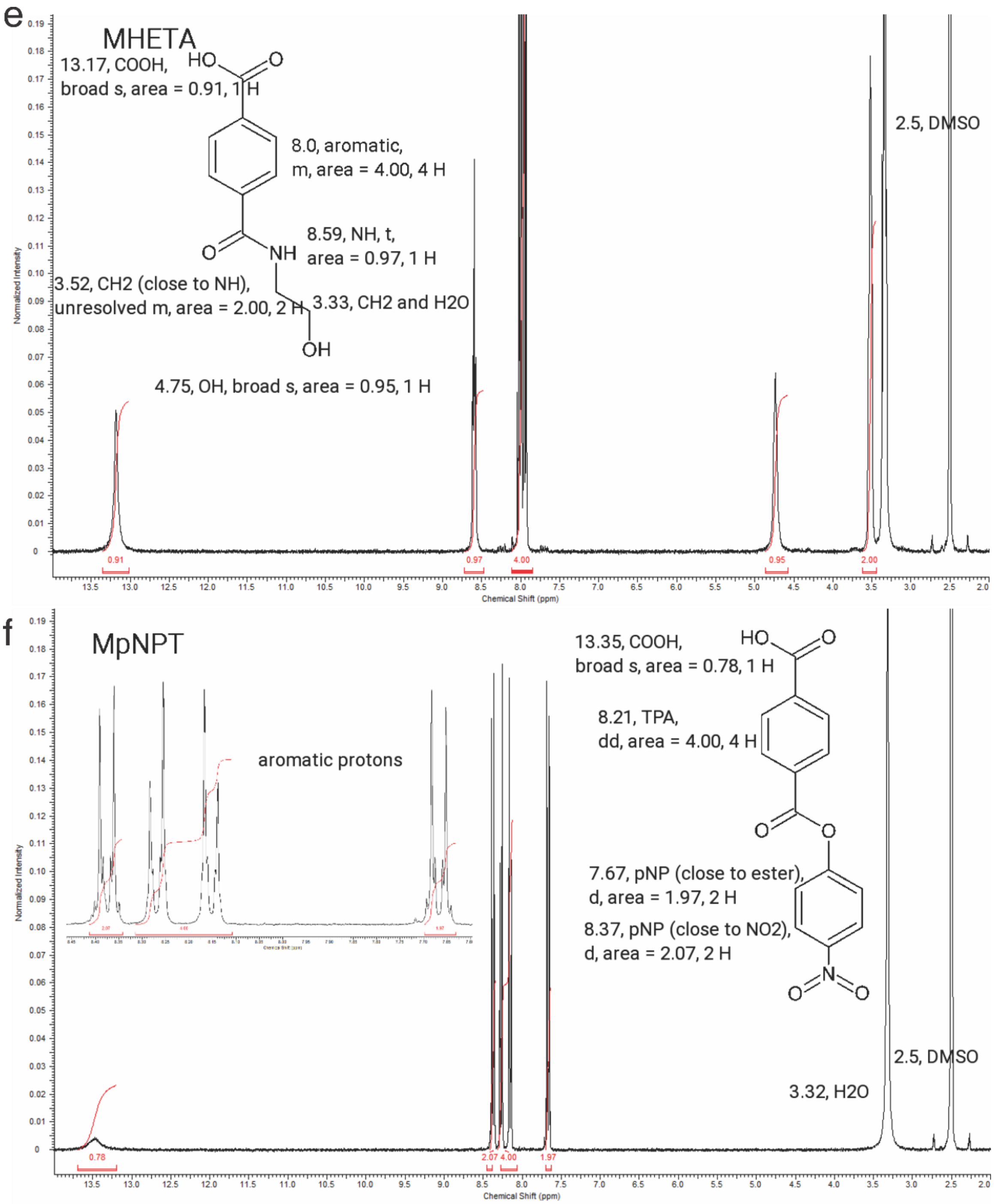

Supplementary Figure 7 ^{1}H NMR spectra of synthesized compounds. All samples were dissolved in DMSO-d_6 resulting in small residual ^{1}H-water and DMSO peaks (3.33 and 2.50 ppm). Chemical shifts are given in ppm relative to TMS, multiplicity is given as s singlet, d doublet, dd doublet of doublets, t triplet, q quadruplet, m multiplet. **(a)** Bis-(2-hydroxyethyl) terephthalate (BHET), **(b)** bis-(2-hydroxyethyl) terephthalic acid amide (BHETA), **(c)** dimethyl terephthalate (DMT), **(d)** mono-(2-hydroxyethyl) terephthalate (MHET), **(e)** mono-(2-hydroxyethyl) terephthalic acid amide (MHETA), **(f)** mono-4-nitrophenyl terephthalate (MpNPT).

Supplementary Figure 8

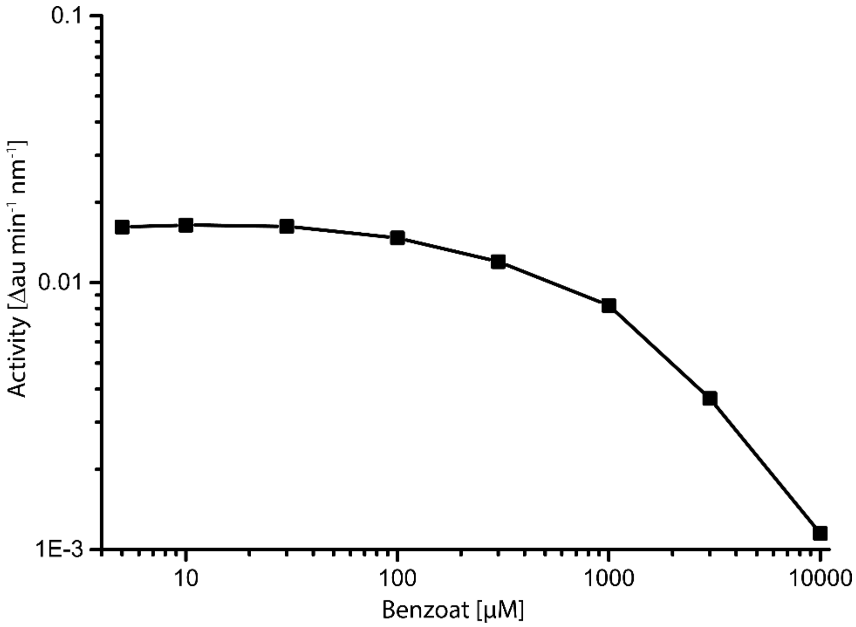

Supplementary Figure 8 Inhibition of MHETase by benzoate. Wild-type MHETase was incubated with different concentrations of benzoate while the hydrolysis rate of MpNPT was quantified. The data was fitted to calculate the K_I value (440 µM in this case). K_I values for different inhibitors and relevant mutants are displayed in Table S2.

Supplementary Figure 9

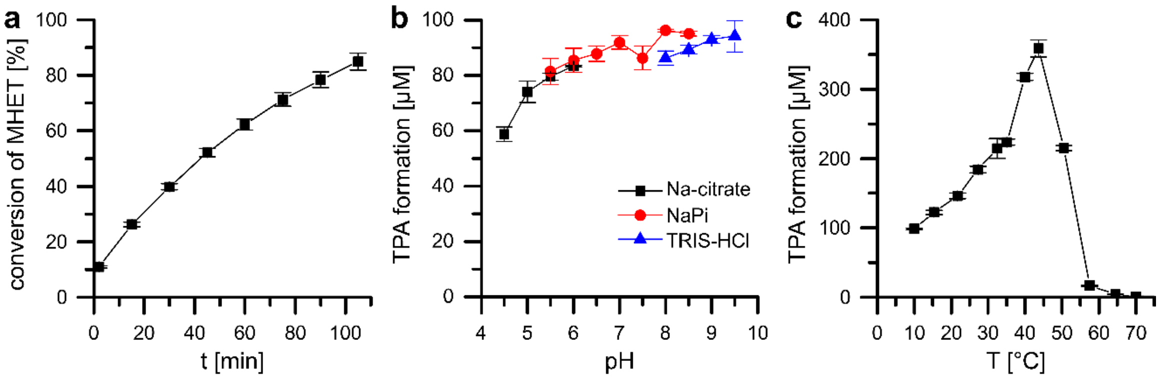

Supplementary Figure 9 Biochemical characterization of MHET conversion and TPA formation dependent on pH and temperature. **(a)** Enzymatic hydrolysis of MHET with MHETase wild type over time. 980 µM MHET was hydrolyzed in 40 mM NaPi pH 7.5 with 80 mM NaCl and 20% (v/v) DMSO by 9.6 nM wild-type MHETase at 30 °C. Decreasing reaction rates at higher turnover of MHET can be explained by product (TPA) inhibition. The data in (a) can be fitted (solid line) with Michaelis-Menten kinetics and competitive inhibition by TPA with K_m = 7 µM, K_i(TPA) = 11.6 µM, k_{cat} = 21.8 s^{-1} = 1310 min^{-1}, $[TPA]_{initial}$ = 93.5 µM. **(b)** Influence of pH. 1 mM MHET was hydrolyzed in 35 mM buffer with 80 mM NaCl and 10% (v/v) DMSO by 4 nM MHETase for 30 min at room temperature. **(c)** Influence of temperature. 1 mM MHET was hydrolyzed in 40 mM NaPi pH 7.5 with 80 mM NaCl and 20% (v/v) DMSO by 8 nM MHETase for 15 min at different temperatures. Error bars represent the standard deviation of triplicates and data points are connected by straight lines for better visualization. TPA was quantified via HPLC in all experiments.

Supplementary Figure 10

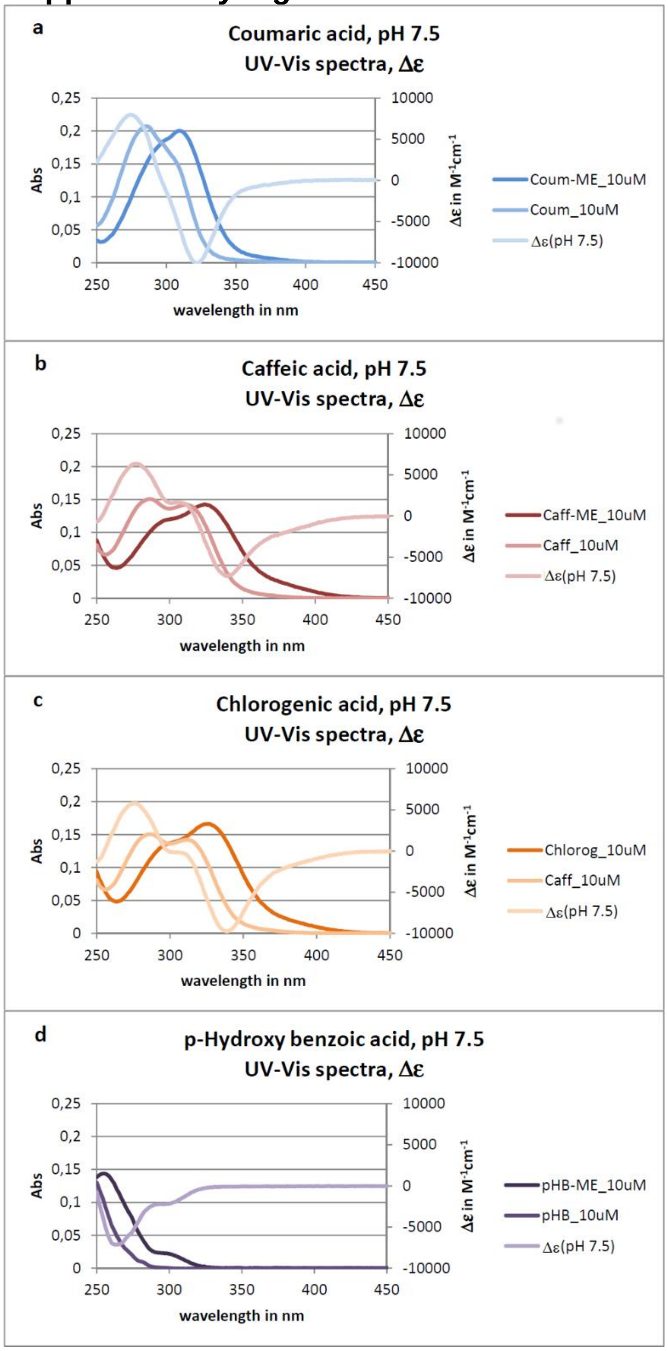

Supplementary Figure 10 Spectra of substrates for feruloyl esterases and tannases. UV-Vis spectra of esters and their corresponding free acids were measured at 10 μM in 100 mM NaPi, pH 7.5. Therefrom, wavelengths were chosen for spectrophotometric activity measurements to have maximal $\Delta\varepsilon = \varepsilon_{product} - \varepsilon_{educt}$ and a reliably measurable absorption for the stronger absorbing initial ester: **(a)** coumaric acid methyl ester (A_{335}(100 μM) = 0.732) and coumaric acid, $\Delta\varepsilon_{335}$ = -6100 $M^{-1}cm^{-1}$, **(b)** caffeic acid methyl ester (A_{350}(100 μM) = 0.715) and caffeic acid, $\Delta\varepsilon_{350}$ = -5700 $M^{-1}cm^{-1}$, **(c)** chlorogenic acid (A_{350}(100 μM) = 0.888) and caffeic acid, $\Delta\varepsilon_{350}$ = -7400 $M^{-1}cm^{-1}$ and **(d)** p-hydroxy benzoic acid methyl ester (A_{280}(100 μM) = 0.496) and p-hydroxy benzoic acid, $\Delta\varepsilon_{280}$ = -3900 $M^{-1}cm^{-1}$.

Supplementary Figure 11

>PETase (*Ideonella sakaiensis*)
CAGACCAATCCGTATGCGCGTGGCCCGAATCCGACCGCGGCGAGCCTGGAAGCGAGCGCGGGTCCG
TTTACCGTGCGTAGCTTTACCGTGAGCCGTCCGAGCGGTTATGGTGCGGGTACCGTTTACTATCCGAC
CAACGCGGGTGGCACCGTGGGTGCGATCGCGATTGTTCCGGGTTATACCGCGCGTCAGAGCAGCATC
AAATGGTGGGGTCCGCGTCTGGCGAGCCACGGTTTCGTGGTTATCACCATTGACACCAACAGCACCCT
GGATCAGCCGAGCAGCCGTAGCAGCCAGCAAATGGCGGCGCTGCGTCAAGTTGCGAGCCTGAACGGT
ACCAGCAGCAGCCCGATCTACGGCAAGGTGGACACCGCGCGTATGGGCGTTATGGGTTGGAGCATGG
GTGGCGGTGGCAGCCTGATTAGCGCGGCGAACAACCCGAGCCTGAAAGCGGCGGCGCCGCAAGCGC
CGTGGGATAGCAGCACCAACTTCAGCAGCGTGACCGTTCCGACCCTGATCTTTGCGTGCGAGAACGAC
AGCATTGCGCCGGTGAACAGCAGCGCGCTGCCGATCTACGATAGCATGAGCCGTAACGCGAAGCAGT
TCCTGGAAATTAACGGTGGCAGCCACAGCTGCGCGAACAGCGGTAACAGCAACCAAGCGCTGATTGG
CAAGAAAGGTGTTGCGTGGATGAAACGTTTTATGGACAACGATACCCGTTATAGCACCTTTGCGTGCGA
AAATCCGAATAGCACCCGTGTGAGCGACTTCCGTACCGCGAACTGCAGC

>MHETase (*Ideonella sakaiensis*)
GGTGGTGGTAGCACCCCGCTGCCGCTGCCGCAACAACAACCGCCGCAACAGGAGCCGCCGCCGCCG
CCGGTTCCGCTGGCGAGCCGCGCGGCGTGCGAGGCGCTGAAGGATGGCAACGGTGACATGGTGTGG
CCGAACGCGGCGACCGTGGTTGAAGTTGCGGCGTGGCGTGATGCGGCGCCGGCGACCGCGAGCGC
GGCGGCGCTGCCGGAGCACTGCGAAGTGAGCGGTGCGATCGCGAAGCGTACCGGCATTGACGGTTA
CCCGTATGAGATCAAATTTCGTCTGCGTATGCCGGCGGAGTGGAACGGCCGTTTCTTTATGGAAGGTG
GCAGCGGTACCAACGGTAGCCTGAGCGCGGCGACCGGTAGCATCGGTGGCGGTCAGATTGCGAGCG
CGCTGAGCCGTAACTTTGCGACCATTGCGACCGATGGCGGTCACGACAACGCGGTGAACGATAACCC
GGATGCGCTGGGTACCGTTGCGTTCGGTCTGGATCCGCAAGCGCGTCTGGACATGGGCTACAACAGC
TATGATCAGGTTACCCAAGCGGGCAAGGCGGCGGTTGCGCGTTTCTACGGTCGTGCGGCGGACAAAA
GCTATTTTATCGGCTGCAGCGAGGGCGGTCGTGAGGGTATGATGCTGAGCCAGCGTTTCCCGAGCCAT
TATGATGGTATTGTGGCGGGTGCGCCGGGTTATCAACTGCCGAAGGCGGGCATTAGCGGTGCGTGGA
CCACCCAAAGCCTGGCGCCGGCGGCGGTGGGCCTGGACGCGCAAGGTGTTCCGCTGATTAACAAAAG
CTTTAGCGACGCGGATCTGCACCTGCTGAGCCAGGCGATCCTGGGTACCTGCGATGCGCTGGACGGC
CTGGCGGATGGTATTGTTGACAACTATCGTGCGTGCCAAGCGGCGTTTGATCCGGCGACCGCGGCGA
ACCCGGCGAACGGTCAGGCGCTGCAATGCGTTGGTGCGAAAACCGCGGACTGCCTGAGCCCGGTGC
AGGTTACCGCGATCAAACGTGCGATGGCGGGTCCGGTTAACAGCGCGGGTACCCCGCTGTACAACCG
TTGGGCGTGGGATGCGGGTATGAGCGGTCTGAGCGGTACCACCTATAACCAGGGCTGGCGTTCCTGG
TGGCTGGGTAGCTTCAACAGCAGCGCGAACAACGCGCAACGTGTGAGCGGTTTCAGCGCGCGTAGCT
GGCTGGTTGACTTCGCGACCCCGCCGGAACCGATGCCGATGACCCAGGTTGCGGCGCGTATGATGAA
GTTCGACTTTGATATCGACCCGCTGAAAATTTGGGCGACCAGCGGCCAGTTCACCCAAAGCAGCATGG
ATTGGCATGGTGCGACCAGCACCGATCTGGCGGCGTTTCGTGACCGTGGCGGTAAAATGATCCTGTAT
CATGGTATGAGCGATGCGGCGTTCAGCGCGCTGGATACCGCGGACTACTATGAACGTCTGGGTGCGG
CGATGCCGGGTGCGGCGGGTTTCGCGCGTCTGTTTCTGGTTCCGGGTATGAACCATTGCAGCGGCGG
TCCGGGTACCGATCGTTTTGACATGCTGACCCCGCTGGTTGCGTGGGTTGAGCGTGGTGAAGCGCCG
GATCAAATCAGCGCGTGGAGCGGTACCCCGGGTTACTTCGGTGTGGCGGCGCGTACCCGTCCGCTGT
GCCCGTATCCGCAAATTGCGCGTTACAAGGGCAGCGGTGACATCAATACCGAAGCGAACTTTGCGTGC
GCGGCGCCGCCG

Supplementary Figure 11 Codon-optimized nucleotide sequences of PETase and MHETase

Supplementary Figure 12

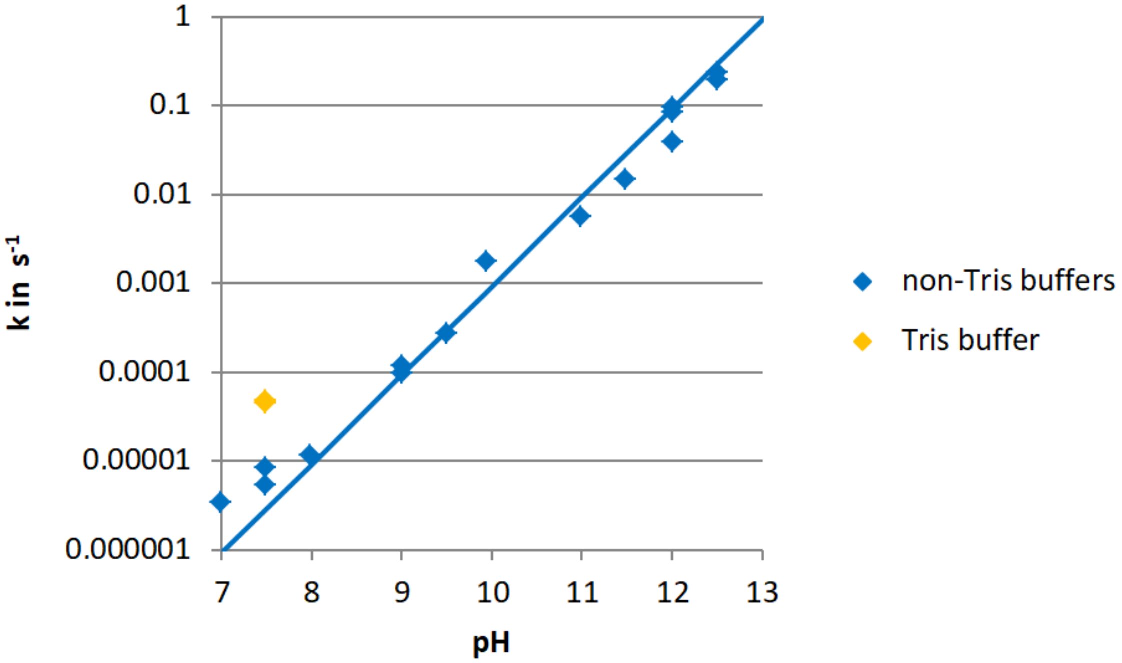

Supplementary Figure 12 Autohydrolysis of MpNPT. $A_{400\ nm}$ was measured for 30 µM MpNPT in 100 mM buffer or NaOH solutions and k_1 fitted. For pH 9 and less final $A_{400\ nm}$ values were taken from complete enzymatic hydrolysis (pH 7: Phosphate: 3.3 * 10^{-6} s^{-1}, pH 7.5: Phosphate: 5.2 * 10^{-6} s^{-1}, 8.1 * 10^{-6} s^{-1}, pH 8: Borate: 11 * 10^{-6} s^{-1}, pH 9: Borate: 93 * 10^{-6} s^{-1}, 120 * 10^{-6} s^{-1}, pH 9.5: Borate: 270 * 10^{-6} s^{-1}, pH 9.93: Borate: 1.7 * 10^{-3} s^{-1}, pH 11: NaOH: 2.7 * 10^{-3} s^{-1}, pH 11.5: NaOH: 15 * 10^{-3} s^{-1}, pH 12: NaOH: 85 * 10^{-3} s^{-1}, 93 * 10^{-3} s^{-1}, 39 * 10^{-3} s^{-1}, pH 12.5: NaOH: 200 * 10^{-3} s^{-1}, 230 * 10^{-3} s^{-1}). Catalysis by H_2O or H^+ might contribute at low pH.

Supplementary Table 1 Data collection, phasing and refinement

Data Collection*	MHETase apo	MHETase-MHETA	MHETase-BA	PETase
**Beamline**	BESSY, 14.1	BESSY, 14.2	BESSY, 14.2	PETRA III, P13
**Wavelength** (Å)	0.9184	0.9184	0.9184	0.9799
**Temperature** (K)	100	100	100	100
**Space Group**	P1	$P2_12_12_1$	$P2_12_12_1$	$P2_1$
**Unit Cell** (Å, Å, Å, °, °, °)	111.29, 137.95, 137.05, 83.01, 66.88, 68.46	112.15, 183.65, 246.75, 90, 90, 90	111.14, 184.05, 247.44, 90, 90, 90	51.12, 78.77, 140.12, 90, 92.56, 90
**Resolution** (Å)	40.00 - 2.05 (2.15 – 2.05)	50.00 - 2.10 (2.22 – 2.10)	50.00 - 2.20 (2.33 – 2.20)	999.00 – 2.00 (2.12 – 2.00)
**Reflections**				
Unique	408240 (38184)	293388 (29728)	254053 (40324)	73090 (11574)
Completeness (%)	93.2 (65.4)	98.9 (93.4)	98.9 (98.0)	97.1 (95.3)
Redundancy	6.3 (4.5)	6.5 (4.9)	4.6 (4.6)	4.1 (3.6)
**I/σ(I)**	5.55 (0.93)	8.85 (1.27)	6.54 (1.13)	8.4 (1.6)
**R_{sym}(I)**[(a)]	0.219 (1.241)	0.195 (1.054)	0.226 (1.334)	0.131 (0.719)
**CC(1/2)**[(b)]	98.6 (45.4)	99.3 (60.2)	98.5 (40.1)	99.1 (62.0)
**Refinement***				
**Resolution** (Å)	47.93 - 2.05 (2.07 -2.05)	49.10 -2.10 (2.1.3 – 2.10)	46.08 – 2.20 (2.24 – 2.20)	48.73 – 2.00 (2.05 – 2.00)
**Reflections**				
Number	408244 (8308)	293303 (11138)	254007 (12502)	69684 (3607)
Completeness (%)	93.3(60.0)	98.9 (84.0)	98.9 (94.0)	97.4 (91.1)
Test set (%)	5.0	1.1	1.1	4.9
**R_{work}**[(c)]	0.210 (0.346)	0.173 (0.308)	0.185 (0.295)	0.214 (0.303)
**R_{free}**[(c)]	0.262 (0.376)	0.198 (0.355)	0.214 (0.346)	0.260 (0.343)
**ESU** (Å)[(d)]	0.32	0.21	0.26	0.16
**Contents of A.U.**[(e)]				
Protein (Molecules)	10	6	6	4
Protein (Residues)	5588	3349	3345	1046
Solvent atoms	3445	2640	1986	454
**Mean B-Factors** ($Å^2$)				
Wilson B	35	28	34	24
Protein	41	28	31	23
Solvent	39	35	35	29
Ligands	48	50	50	39
**Ramachandran Plot**[(f)]				
Favored (%)	95.30	96.81	97.02	97.50
Outliers (%)	0.23	0.30	0.03	0
**Rmsd**[(g)]				
Bond Lengths (Å)	0.011	0.010	0.007	0.016
Bond Angles (°)	1.117	1.040	0.707	1.817

*) highest resolution shell in parentheses

(a) $R_{sym}(I) = \Sigma_{hkl}\Sigma_i |I_i(hkl) - \langle I(hkl)\rangle| / \Sigma_{hkl}\Sigma_i |I_i(hkl)|$; for n independent reflections and i observations of a given reflection; <I(hkl)> – average intensity of the i observations

(b) Correlation factor CC(1/2) between random half-datasets for reporting results in XDS CORRECT and XSCALE[7].

(c) $R = \Sigma_{hkl} ||F_{obs}| - |F_{calc}|| / \Sigma_{hkl} |F_{obs}|$; R_{work} – hkl $\notin$ T; R_{free} – hkl $\in$ T; R_{all} – all reflections;

(d) ESU – estimated overall coordinate error based on maximum likelihood

(e) A.U. – asymmetric unit

(f) Calculated with phenix[8]

(g) Rmsd – root-mean-square deviation from target geometry

Supplementary Table 2 Inhibition of MHETase and mutants by different inhibitors. Wild-type and mutant MHETase were incubated with different concentrations of inhibitors while the hydrolysis rate of MpNPT was quantified. The data was fitted to calculate the K_I values. Supplementary Figure S8 shows the data for the wild-type enzyme and benzoate as an example.

Variant	Substrate	Inhibitor	K_I in µM
wt	MpNPT	benzoate	440
wt	MpNPT	benzamide	>5000
wt	MpNPT	terephthalate	317
R411Q	MpNPT	benzoate	>5000
R411Q	MpNPT	benzamide	>5000
R411Q	MpNPT	terephthalate	3881
S416A	MpNPT	benzoate	2906
S416A	MpNPT	benzamide	1910
S416A	MpNPT	terephthalate	2560

Supplementary Table 3 Compounds used in this study and their numbering.

No.	Compound name
0	no ligand
1	benzoic acid amide
2	benzoic acid methyl ester
3	bis-(2-hydroxyethyl) terephthalic acid (BHET)
4	bis-(2-hydroxyethyl) terephthalamide (BHETA)
5	nicotinic acid amide
6	4-hydroxybenzoic acid methyl ester
7	4-nitrobenzoic acid methyl ester
8	terephthalic acid dimethyl ester
9	4-nitrophenol, sodium salt
10	4-nitrothiophenol
11	4-toluene sulfonic acid
12	benzene arsonic acid
13	3-methylbenzoic acid
14	2-methylbenzoic acid
15	4-methylbenzoic acid
16	nicotinic acid
17	terephthalic acid
18	benzoic acid
19	4-hydroxybenzoic acid
20	mono-(2-hydroxyethyl) terephthalamide (MHETA)
21	mono-(2-hydroxyethyl) terephthalic acid (MHET)

Supplementary Table 4 Oligonucleotides for site-directed mutagenesis of the MHETase gene

Primer name	Sequence 5' ⟶ 3'
H488A_fw	GATCCTGTATGCGGGTATGAGCGATGCGGC
H488A_rv	ATTTTACCGCCACGGTCA
S416A_fw	GAGCGGTTTCGCGGCGCGTAGCTG
S416A_rv	ACACGTTGCGCGTTGTTC
F415A_fw	TGTGAGCGGTGCGAGCGCGCGTAG
F415A_rv	CGTTGCGCGTTGTTCGCG
F415H_fw	TGTGAGCGGTCATAGCGCGCGTAG
F415H_rv	CGTTGCGCGTTGTTCGCG
F495A_fw	CGATGCGGCGGCGAGCGCGCTGG
F495A_rv	CTCATACCATGATACAGGATCATTTTACCG
W397A_fw	CTGGCGTTCCGCGTGGCTGGGT
W397A_rv	CCCTGGTTATAGGTGGTAC
S416G_fw	GAGCGGTTTCggcGCGCGTAGCT
S416G_rev	ACACGTTGCGCGTTGTTCGC
R411Q_S416G_fw	GAGCGGTTTCgGCGCGCGTAG
R411Q_S416G_rev	ACTTGTTGCGCGTTGTTCGC
R411Q_S416A_fw	GAGCGGTTTCgcCGCGCGTAGC
R411Q_S416A_rev	ACTTGTTGCGCGTTGTTC
R411A_S416G_fw	GAGCGGTTTCgGCGCGCGTAG
R411A_S416G_rev	ACTGCTTGCGCGTTGTTCG
R411A_S416A_fw	GAGCGGTTTCgcCGCGCGTAGC
R411A_S416A_rev	ACTGCTTGCGCGTTGTTC
S419G_fw	CAGCGCGCGTgGCTGGCTGGT
S419G_rev	AAACCGCTCACACGTTGCGCG
F424N_fw	GCTGGTTGACaaCGCGACCCCG
F424N_rev	CAGCTACGCGCGCTGAAA
F424Q_fw	GCTGGTTGACcagGCGACCCCGC
F424Q_rev	CAGCTACGCGCGCTGAAA
R411A_S419G_fw	CAGCGCGCGTgGCTGGCTGGT
R411A_S419G_rev	AAACCGCTCACTGCTTGCGCG
R411A_fw	CGAACAACGCGCAAGCTGTGAGCGGTTTC
R411A_rev	GAAACCGCTCACAGCTTGCGCGTTGTTCG
QC_S225A_fw	CTATTTTATCGGCTGCGCCGAGGGCGGTCGTGAG
QC_S225A_rv	CTCACGACCGCCCTCGGCGCAGCCGATAAAATAG
QC_D492A_fw	CATGGTATGAGCGCTGCGGCGTTCAG
QC_D492A_rv	CTGAACGCCGCAGCGCTCATACCATG
QC_H528A_fw	GTTCCGGGTATGAACGCTTGCAGCGGCGGTC
QC_H528A_rv	GACCGCCGCTGCAAGCGTTCATACCCGGAAC
QC_R411Q_fw	GAACAACGCGCAACAGGTGAGCGGTTTCAG
QC_S419G_S416G_R411A_fw	GTTTCGGCGCGCGTGGATGGCTGGTTGACTTC
QC_S419G_S416G_R411A_rev	GAAGTCAACCAGCCATCCACGCGCGCCGAAAC
F424N_ S416A_fw	GCTGGTTGACaaCGCGACCCCG
F424N_S416A_rev	CAGCTACGCGCCGCGAAA
F424N_S419G_rev	CAGCCACGCGCGCTGAAA
S416A_S419G _fw	GAGCGGTTTCgcCGCGCGTGGC
S416A_S419G _rev	ACACGTTGCGCGTTGTTC
H467N_F424N_fw	CATGGATTGGaATGGTGCGAC
H467N_F424N_rev	CTGCTTTGGGTGAACTGG
L254N_F424N_fw	GGGTTATCAAaacCCGAAGGCGGGCATTAGCG
L254N_F424N_rev	GGCGCACCCGCCACAATA
F424A_fw	GCTGGTTGACgcCGCGACCCCG
F424A_rev	CAGCTACGCGCGCTGAAA
F424S_fw	CTGGTTGACTcCGCGACCCCG
F424S_rev	CCAGCTACGCGCGCTGAA
F424N_F415H_fw	GCTGGTTGACaaCGCGACCCCG
F424N_F415H_rev	CAGCTACGCGCGCTATGA

Supplementary References

1. Suzuki, K. *et al.* Crystal structure of a feruloyl esterase belonging to the tannase family: A disulfide bond near a catalytic triad. *Proteins Struct. Funct. Bioinforma.* **82,** 2857–2867 (2014).
2. Ren, B. *et al.* Crystal structure of tannase from *Lactobacillus plantarum*. *J. Mol. Biol.* **425,** 2737–2751 (2013).
3. Larkin, M. A. *et al.* Clustal W and Clustal X version 2.0. *Bioinformatics* **23,** 2947–2948 (2007).
4. Barton, G. J. Alscript: A tool to format multiple sequence alignments. *Protein Eng. Des. Sel.* **6,** 37–40 (1993).
5. Mueller, U. *et al.* Facilities for macromolecular crystallography at the Helmholtz-Zentrum Berlin. *J. Synchrotron Radiat.* **19,** 442–449 (2012).
6. Han, X. *et al.* Structural insight into catalytic mechanism of PET hydrolase. *Nat. Commun.* **8,** 2106 (2017).
7. Karplus, P. A. & Diederichs, K. Linking Crystallographic Model and Data Quality. *Science.* **336,** 1030–1033 (2012).
8. Adams, P. D. *et al.* PHENIX: A comprehensive Python-based system for macromolecular structure solution. *Acta Crystallogr. Sect. D Biol. Crystallogr.* **66,** 213–221 (2010).
